# Semaphorin4B is elevated in rheumatoid arthritis and enhances the inflammatory phenotype of macrophages and fibroblast-like synoviocytes

**DOI:** 10.1186/s13075-025-03592-x

**Published:** 2025-07-01

**Authors:** Sara Martínez-Ramos, Carlos Rafael-Vidal, Jaime Marty, Beatriz Malvar-Fernández, Coral Mouriño, Nair Pérez, Irene Altabás, Francisco J. Maceiras Pan, David Fernández-Fernández, Carmen Conde, Megan M. Hanlon, Conor M. Smith, Paul Peter Tak, Douglas J. Veale, Ursula Fearon, Jose María Pego-Reigosa, Samuel García

**Affiliations:** 1 Rheumatology & Immuno-mediated Diseases Research Group (IRIDIS), Galicia Sur Health Research Institute (IIS Galicia Sur), SERGAS-UVIGO, Vigo, Spain; 2 Rheumatology Department, University Hospital Complex of Vigo, Vigo, Spain; 3 Laboratorio de Reumatologia Experimental y Observacional y Servicio de Reumatologia, Instituto de Investigacion Sanitaria de Santiago (IDIS), Hospital Clinico Universitario de Santiago de Compostela (CHUS), Servizo Galego de Saude (SERGAS), Santiago de Compostela, Spain; 4 Molecular Rheumatology, Clinical Medicine, Trinity Biomedical Science Institute, Dublin, Ireland; 5 EULAR Centre for Arthritis and Rheumatic Diseases, St Vincent’s University Hospital, University College Dublin, Dublin, Ireland; 6 Translational Immunology, Trinity Biomedical Sciences Institute, Trinity College Dublin, Dublin, Ireland; 7 Amsterdam Rheumatology and Immunology Center, Academic Medical Center of the University of Amsterdam, Amsterdam, The Netherlands; 8 Candel Therapeutics, Needham, MA USA

**Keywords:** Semaphorin4B, Rheumatoid arthritis, Synovium, Fibroblast-like synoviocytes, Macrophages, Inflammation

## Abstract

**Background:**

Several members of the class 4 semaphorins are involved in the pathogenesis of rheumatoid arthritis (RA), regulating proinflammatory functions, but the role of (Sema)phorin4B remains unexplored. Therefore, the aim of this study was to determine the expression and function of Sema4B in RA.

**Methods:**

Peripheral blood monocytes from healthy controls (HC) and patients with RA were differentiated into M1 macrophages and stimulated with Sema4B and LPS alone and in combination. Fibroblast-like synoviocytes (FLS) from patients with osteoarthritis (OA) and RA and 3D synovium micromasses, formed by RA FLS and RA monocytes, were stimulated with Sema4B and TNF-α alone and in combination. PlexinB2 expression was knocked down using siRNA. *Synovial* mRNA expression was obtained from gene expression array in GEO-NCBI and determined by (q)uantitative PCR. Protein expression was determined by immunohistochemistry, immunoblotting and ELISA. FLS viability, *invasion* and migration were determined using calcein, *invasion and* wound repair scratch assays, respectively.

**Results:**

The expression of Sema4B was higher in the synovial tissue of patients with RA, as well as in RA FLS compared to OA FLS. Importantly, the stimulation of RA FLS and RA MØ with inflammatory mediators induced the expression of Sema4B. Functionally, Sema4B alone induced FLS migration *and invasion*, and enhanced the TNF-α or LPS-induced production of inflammatory mediators (Interleukin (IL)-6, IL-8, TNF-α, CCL-2) and matrix metalloproteases (MMP-1 and MMP-3) in RA FLS, RA MØ and the 3D synovium model. In RA FLS, this effect was mediated by the receptor PlexinB2.

**Conclusions:**

In an inflammatory context, Sema4B induces *an aggressive* FLS *phenotype* and the production of pro-inflammatory mediators by FLS and MØ. These results suggest that Sema4B is involved in the *pathogenic* processes observed within the RA synovium.

**Supplementary Information:**

The online version contains supplementary material available at 10.1186/s13075-025-03592-x.

## Introduction

The semaphorin family is a large group of proteins that are involved in the pathogenesis of rheumatoid arthritis (RA) [1]. While the expression of class 3 semaphorins is reduced in patients with RA and they play protective roles in the disease [2–4], members of class 4 have revealed effector functions. Synovial fluid and serum levels of Sema4A are elevated in patients with RA and positively correlate with the clinical activity scores [5, 6]. Functionally, Sema4A promotes inflammation and enhances the invasive ability of RA FLS [5]. The systemic and local expression of Sema4D is also higher in patients with RA and the serum levels correlate with the DAS28 score. Moreover, Sema4D induces the production of inflammatory mediators by RA monocytes, while the administration of an anti-Sema4D antibody reduces the arthritis severity and inflammatory cytokines in the Collagen Induced Arthritis (CIA) mouse model [7].

Sema4B is another member of class 4 semaphorins that is involved in the induction of cell proliferation and invasion [8, 9], neuronal cell proliferation and survival [10], and microglia/macrophage activation [11, 12], contributing to the pathogenesis of several diseases, including lung cancer and brain injury. Similarly to other members of the class 4 semaphorins, Sema4B binds to class B plexin receptors, specifically to PlexinB2 [11]. But Sema4B also can bind to other receptors such as Discoidin, CUB and LCCL domain containing 2 (DCBLD2) [13] and Discs large MAGUK scaffold protein 4 (DLG4) [14].

Despite the pathogenic functions of Sema4B in other diseases, and in contrast to other class 4 semaphorins, the involvement of Sema4B in the pathogenesis of RA remains unexplored. Therefore, the aim of this study was to determine the expression of Sema4B in patients with RA and the potential role in the pathogenesis of the disease.

## Methods

### Patients

FLS were obtained from patients with RA and knee osteoarthritis (OA) by needle arthroscopy, as previously described [15]. Peripheral blood was acquired from patients with RA and healthy controls (HC). Patients with RA fulfilled the American College of Rheumatology (ACR)/European Alliance of Associations for Rheumatology (EULAR) 2010 criteria for RA [16], while patients with OA grade 2–4, according to the radiological criteria of Kellgren and Lawrence [17], were included in this study. Prior to inclusion in this study, all subjects provided written informed consent approved by the Ethics Committee of Galicia (study number 2023/145). Clinical characteristics of all patients are detailed in supplementary Table S1.

### Gene expression from profiling data

The gene expression of *SEMA4B*,* PLXNB2*, *DCBLD2* and *DLG4* was retrieved from array profiling data available at the Gene Expression Omnibus (GEO–NCBI) GSE7729 [18], GSE48780 [19], GSE95588 [20] and GSE200815 [21, 22].

### Monocyte purification, macrophage differentiation and stimulation

PBMCs were obtained by using Ficoll gradient (STEMCELL Technologies) and CD14^+^ monocytes were isolated with the MagniSort Human pan-Monocyte isolation kit (Thermo Fisher Scientific). Monocytes were differentiated into M1 MØ by culturing in Iscove’s Modified Dulbecco’s Medium (IMDM, Lonza™) supplemented with 10% of heat-inactivated fetal bovine serum (FBS, Corning™) and 10,000 U/mL penicillin-streptomycin, in the presence of IFN-γ (10 ng/mL, R&D Systems). At day 6, RA MØ were stimulated with rhSema4B (200 ng/mL, R&D Systems), alone or in combination with LPS (10 ng/mL, InvivoGen) for 24 h.

### FLS culture and stimulation

FLS were cultured in Dulbecco’s Modified Eagle Medium (DMEM) supplemented with 10% FBS, 200 mM Glutamine and 10,000 U/mL penicillin-streptomycin (all Lonza™ BioWhittaker™), and used between passages 6 to 10. When 90% confluence was reached, non-stimulated cells were lysed in for mRNA/protein expression analysis. Additionally, after overnight starvation in DMEM containing 1% FBS, RA FLS were stimulated with recombinant human (rh)Sema4B (200 ng/mL) alone or in combination with TNF-α (10 ng/mL, R&D Systems) or IL1-β (1 ng/mL, R&D Systems) for 4 and 24 h.

### SiRNA transfection

RA FLS were transfected using DharmaFECT 1 (Horizon Discovery). RA FLS were incubated in DMEM with 10% FBS and without antibiotic for 6 h at 37 °C. Silencing was performed with Sema4B, PlexinB2 and control non-targeting siRNAs (50 nM, Horizon Discovery) in Opti-MEM (Thermo Fisher Scientific) for 24 h. Opti-MEM was then replaced by DMEM with 10% FBS. After 24 h, cells were serum-deprived and stimulated as already detailed. Efficiency of transfections is shown in Supplementary Figure S1.

### RT-PCR and quantitative (q)PCR

RNA from FLS, MØ and micromasses was isolated by using the NucleoSpin RNA/Protein Mini kit (Macherey-Nagel). Reverse-transcription of total RNA was conducted with iScript (Biorad), while cDNA was amplified by qPCRs in duplicates using SYBR green (Biorad) and specific primers (Integrated DNA Technologies IDT; supplementary Table S2) within a CFX96 Touch Real-Time PCR Detection System (Biorad). Relative gene expression was normalized to the expression of 3 housekeeping genes (*GAPDH*, *B2M* and *RPL13*). mRNA relative expression was calculated by using the formulas *2*^*–ΔCt*^*x 1000* or *2*^*–ΔΔCt*^ (relative quantity, RQ), as applicable.

### Measurement of cytokine production

IL-6 and TNF-α protein levels were assessed by ELISA (both R&D Systems) in cell-free supernatants, according to manufacturing instructions.

### Immunoblotting

FLS and MØ were lysated in Laemmli buffer. Equal quantities of total protein were fractionated by 10% Polyacrylamide gels and transferred to PVDF Transfer membranes (Thermo Fisher Scientific). Membranes were incubated at 4 ºC overnight with primary antibodies for Sema4B (GeneTex), tubulin (Tub, R&D Systems) and β-actin (R&D Systems) in 4% Milk-TBS/T, washed and incubated in 2% Milk-TBS/T containing HRP-conjugated secondary antibody (anti-mouse IgG, Thermo Fisher Scientific). Protein was developed with ECL Western Blotting Substrate (Thermo Fisher Scientific) employing a ChemiDocTM MP System (Biorad). Densitometry analysis was performed by ImageJ software and relative protein expression was normalized to tubulin or β-actin expression.

### Immunohistochemistry

Sections of paraffin-embedded biopsy samples from HC (*n* = 5) and patients with RA (*n* = 7) were cut serially with a microtome (8 μm). Deparaffinization, re-hydration and epitope-retrieval processes were performed in citrate buffer (pH 6.0) using a PT Link station (Agilent). Samples were treated with peroxidase inhibitor (Roche), blocked with 5% BSA/PBS and incubated overnight at 4 ºC with primary antibody for Sema4B (25 µg/mL, GeneTex) and IgG2A (25 µg/mL, Abcam) as negative control, both diluted in 5% BSA/PBS. Sections were then washed and incubated with HRP-conjugated secondary antibody (anti-rabbit IgG, R&D systems), and consecutively with hydrogen peroxide and DAB (both Roche). The staining was performed with haematoxylin solution (Sigma-Aldrich) and mounted in Mounting Medium (ITW Reagents).

### Viability assay

5,000 RA FLS per well were seeded in 96-well plates and stimulated with rhSema4B (200 ng/mL) for 24, 48, 72 and 96 h. After incubation with Calcein-AM (1 µM, Thermo Fisher Scientific) for 2 h, fluorescence was measured in a FLUOstar Omega microplate reader (excitation range 490 nm, emission range 520 nm).

### Migration assay

RA FLS migration was evaluated by the wound healing assay. A straight scratch was made on confluent plated FLS with a 200-µL tip. Wells were then washed to remove unattached cells and stimulated with Sema4B (200 ng/mL) in 1% or 10% FBS-containing DMEM. Light microscopy images were taken immediately (time point 0) and after 24 h stimulation. The number of migrated cells was averaged from three 10-field-of-view images and normalized to non-stimulated conditions.

### Invasion assays

The permeable supports of 24-well plates with 8 µM transparent PET membranes (Corning) were coated with Corning^®^ Matrigel^®^ Growth Factor Reduced (GFR) Basement Membrane Matrix (2 mg/ml, Corning). To measure cell invasion, 3 × 10^4^ FLS in medium containing 1% FBS were added to the transwells. Sema4B (200 ng/ml) was used as an attractant in the lower chamber. After 48 h, the cells that invaded through the matrix were fixed and stained with crystal violet 0.5%. The number of invading cells was averaged from 3 10-field-of-view images and normalized to unstimulated cells.

### 3D synovium micromasses

2 × 10^4^ RA FLS and 6 × 10^4^RA-derived CD14^+^ monocytes were mixed on ice with 25 µL liquid Matrigel^®^ (Corning) and seeded on a poly-2-hydroxyethyl methacrylate (poly-HEMA, Sigma-Aldrich) coated 24-well plate. Micromasses were cultured at 37 °C and 5% CO_2_ in IMDM supplemented with 10% FBS and 10,000 U/mL penicillin-streptomycin in the presence of TNF (10 ng/mL), rhSema4B (200 ng/mL) or the combination of both. Medium was replaced at day 3 and, at day 7, micromasses were lysed RNA isolation.

### Statistical analyses

Statistical analysis was performed by using Windows GraphPad Prism 8 (GraphPad Software, Inc.). Normality was analysed by Shapiro-Wilk and Kolmogorov-Smirnov tests. The potential differences between experimental groups following normal distribution were analysed by Paired t test, Wilcoxon test, One-way ANOVA, Friedman test and Two-way ANOVA, as applicable. Data non-following normal distribution were analysed by Mann-Whitney test or Friedman’s test, as appropriate. *P* values ≤ 0.05 were considered statistically significant.

## Results

### Sema4B expression is elevated in the synovium of patients with RA

**Fig. 1:**
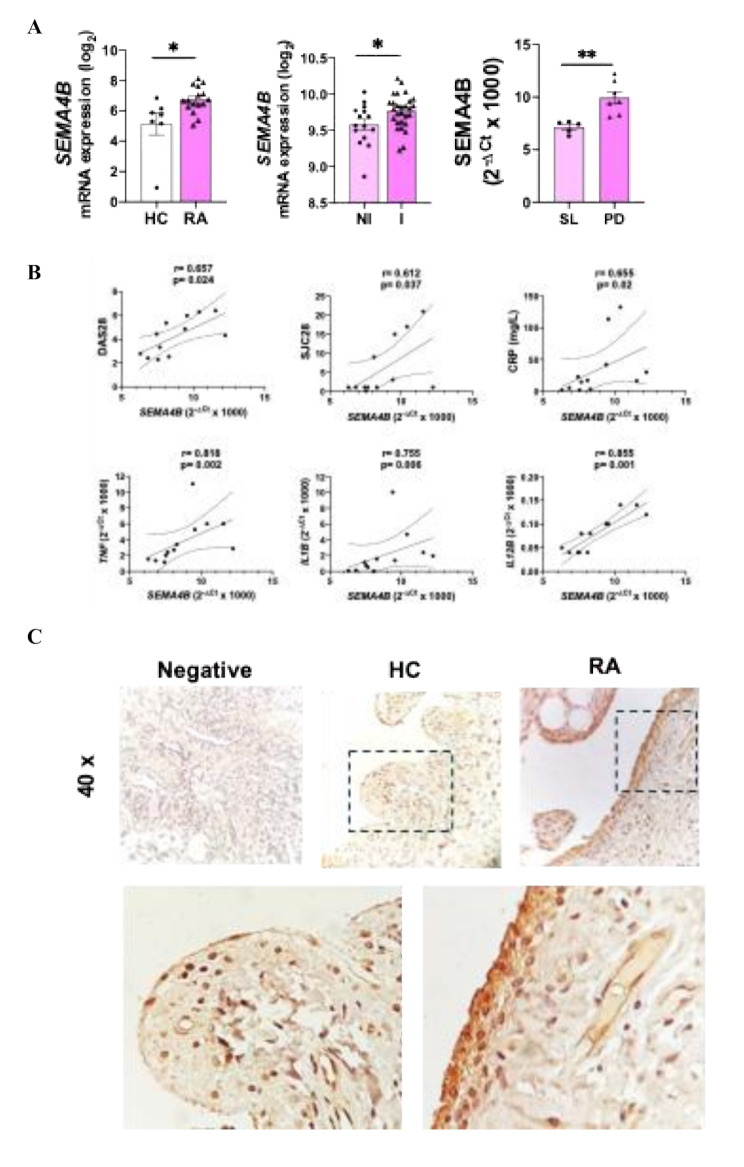
Sema4B expression is elevated in the synovium of patients with RA. **(A)***SEMA4B* expression in the synovium of healthy controls (HC, *n* = 7) and patients with RA (*n* = 17), in the inflamed (I, *n* = 15) and non-inflamed (NI, *n* = 28) RA synovium or in the synovium of self-limiting (SL, *n* = 5) or persisted diseases (PD, *n* = 7) RA patients. **(B)** Correlation analysis of *SEMA4B* with clinical disease parameters and the expression of inflammatory mediators in the synovial tissue of early, DMARD-näive arthritis patients. **(C)** Representative slides of Sema4B expression in the synovial tissue of patients with RA (*n* = 7) and HC (*n* = 5). Data are shown as log_2_ of Fragments Per Kilobase per Million Mapped Fragments (FPKM) or as 2^−ΔCt^ x1000. *p < 0.05 and **p < 0.01

We first determined the expression of Sema4B in the synovial tissue of HC and patients with RA, using public available datasets. *SEMA4B* was significantly increased in patients with RA compared to HC. Importantly, within the RA synovium, *SEMA4B* expression was significantly higher in the inflamed tissue compared to the non-inflamed sections. Moreover, in the synovial tissue of 12 early (disease duration < 1 year), DMARD-naïve RA patients, previously used by our group [23], *SEMA4B* expression was elevated in patients who had had persistent disease after 2 years’ follow-up, compared to patients who had self-limiting disease (Fig. 1A). In these patients, SEMA4B positively and significantly correlated with clinical disease parameters -DAS28-, swollen joint count -SJC- and C-reactive protein -CRP- levels) and the expression of the inflammatory mediators significantly higher in the inflamed tissue *TNF*, *IL1B* and *IL12B* (Fig. 1B).

To confirm these findings, IHC analysis of the synovial tissue from HC and patients with RA was performed and we found that the expression of Sema4B was elevated in patients with RA compared to HC. Interestingly, Sema4B was mainly expressed in the intimal lining layer, but also some expression was observed in the sublining (Fig. 1C).

**Fig. 2:**
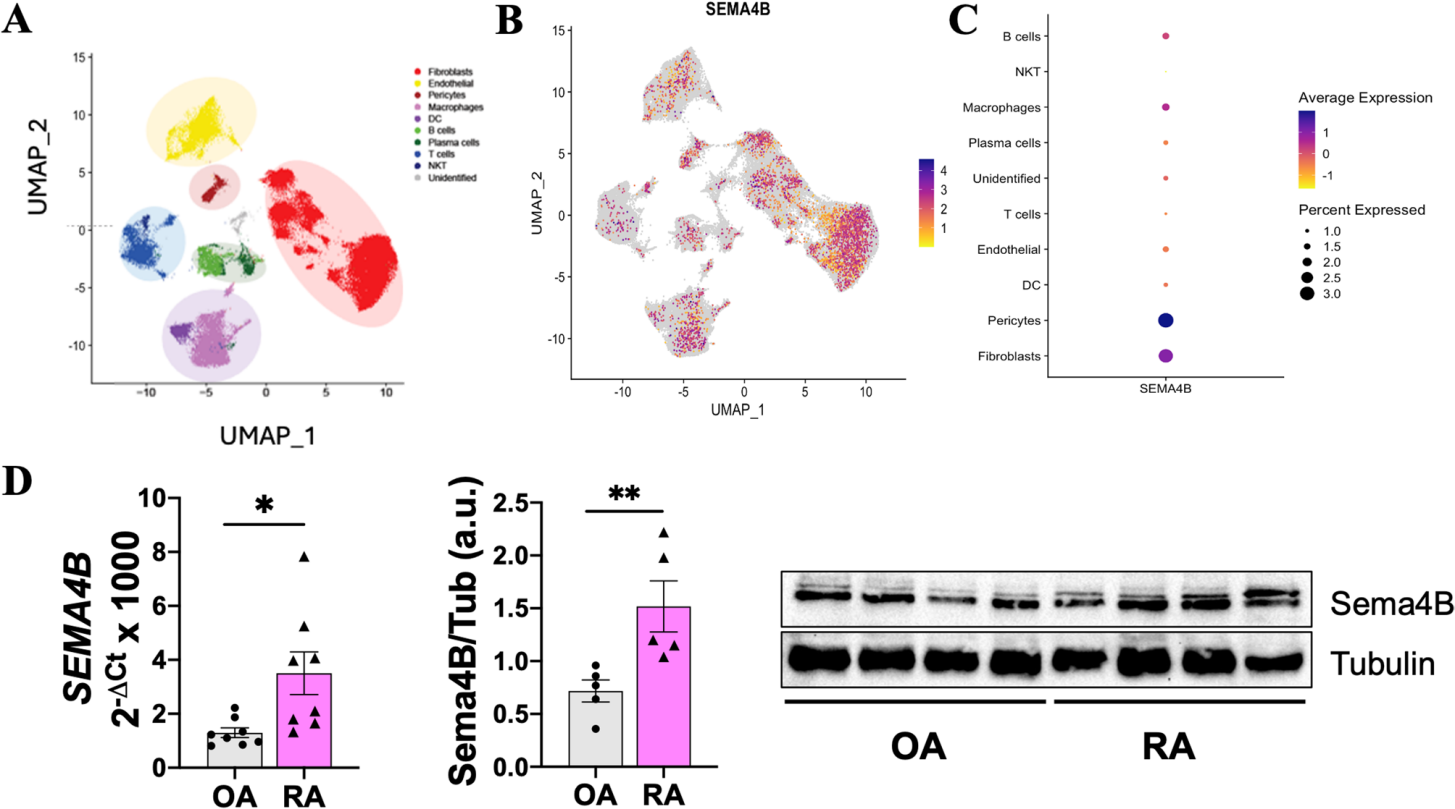
Sema4B is expressed by RA FLS and macrophages. **(A)** High dimensionality single cell RNA sequencing analysis identifies specific cell clusters in patient with RA synovial tissue biopsies. **(B)** Feature plots for the expression and distribution of *SEMA4B* in all cells. **(C)** Expression and percentage of positive cells per cell population for *SEMA4B*. **(D)** mRNA expression, densitometric analysis and representative immunoblot of Sema4B in RA and OA FLS (*n* = 5–8). *p < 0.05 and **p < 0.01

To determine the cells that express Sema4B within the RA synovium, analysis of a public available scRNAseq database. The highest expression of Sema4B was observed in FLS, macrophages and pericytes (Fig. 2A-C). Interestingly, macrophage *SEMA4B* expression was mostly enriched in a specific cluster (6), which is a TREM2^+^VSIG4^+^ macrophage population considered as a tissue resident macrophage [22] (Supplementary Figure S2A-C). Since FLS and MØ are the predominant cells in the RA synovium and play essential functions in the pathogenesis of the disease, we focused in these 2 cell populations [24]. Sema4B expression by RA FLS was significantly higher compared to OA FLS, at both the mRNA and the protein level (Fig. 2D). However, no differences in SEMA4B levels were observed between RA and HC MØ (Supplementary Figure S2D).

**Fig. 3:**
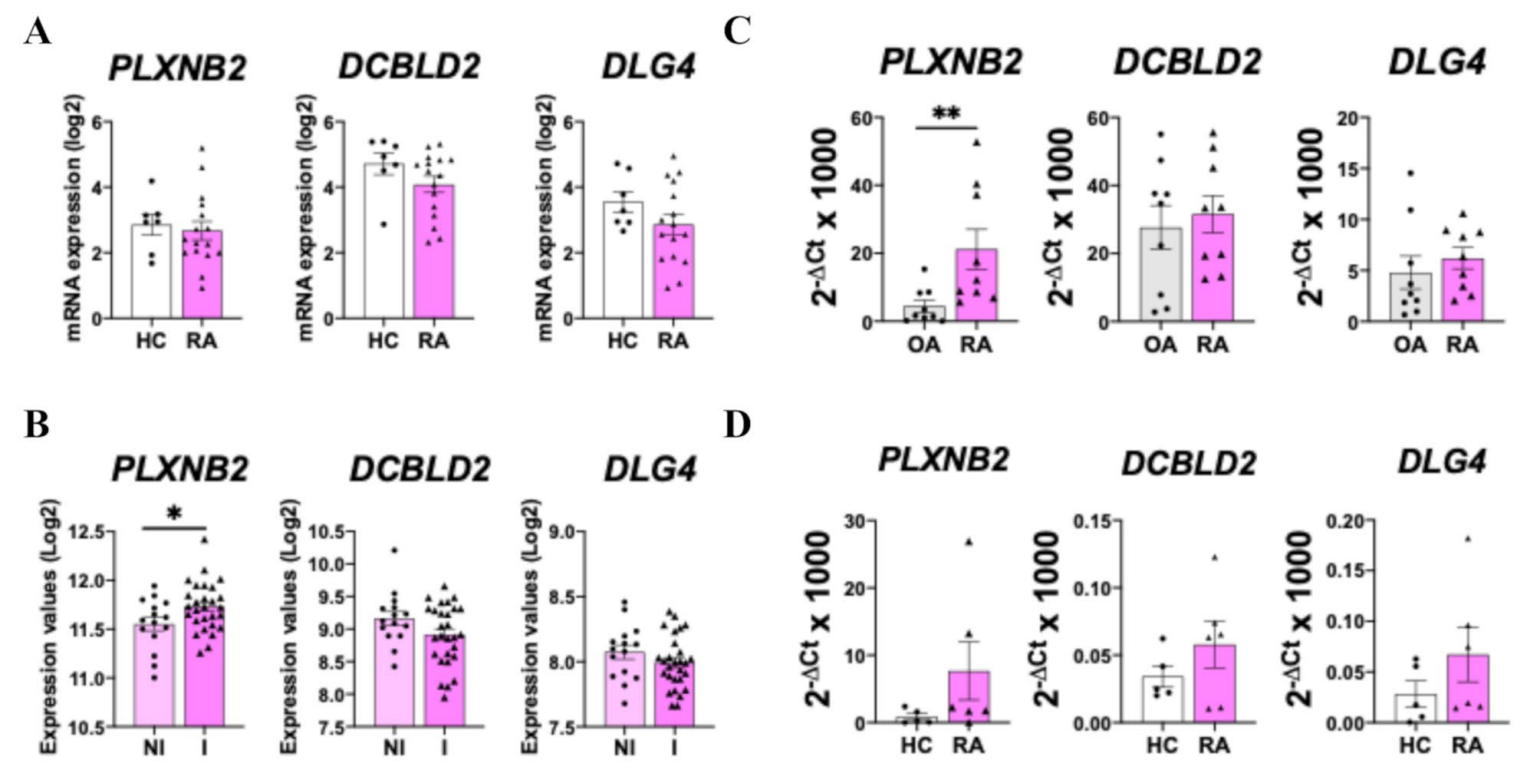
PlexinB2 expression is elevated in the synovium of patients with RA. Expression of Sema4B receptors expression in the synovium of healthy controls (HC, *n* = 7) and patients with RA (*n* = 17) (**B**), in the inflamed (I, *n* = 15) and non-inflamed (NI, *n* = 28) RA synovium (**C**), in RA and OA FLS (*n* = 9) (**C**) and in HC and RA macrophages (*n* = 5). Data are shown as log_2_ of Fragments Per Kilobase per Million Mapped Fragments (FPKM) or as 2^−ΔCt^ x1000. *p < 0.05 and **p < 0.01

The expression of the Sema4B receptors in the synovium of patients with RA was also determined. *PLXNB2*, *DCBLD2* and *DLG4* expression was similar between HC and patients with RA, although the levels of *PLXNB2* were elevated in the inflamed sections of RA synovium (Fig. 3A-B). In line with these findings, the expression of *PLXNB2* was also significantly higher in RA compared to OA FLS, while the expression of the other receptors was similar (Fig. 3C). Results also showed a slight increase of *PLXNB2*, *DCBLD2* and *DLG4* levels in RA MØ compared to HC MØ, but differences were not significant (Fig. 3D).

### Sema4B expression is induced by inflammatory mediators in RA FLS and RA macrophages

**Fig. 4:**
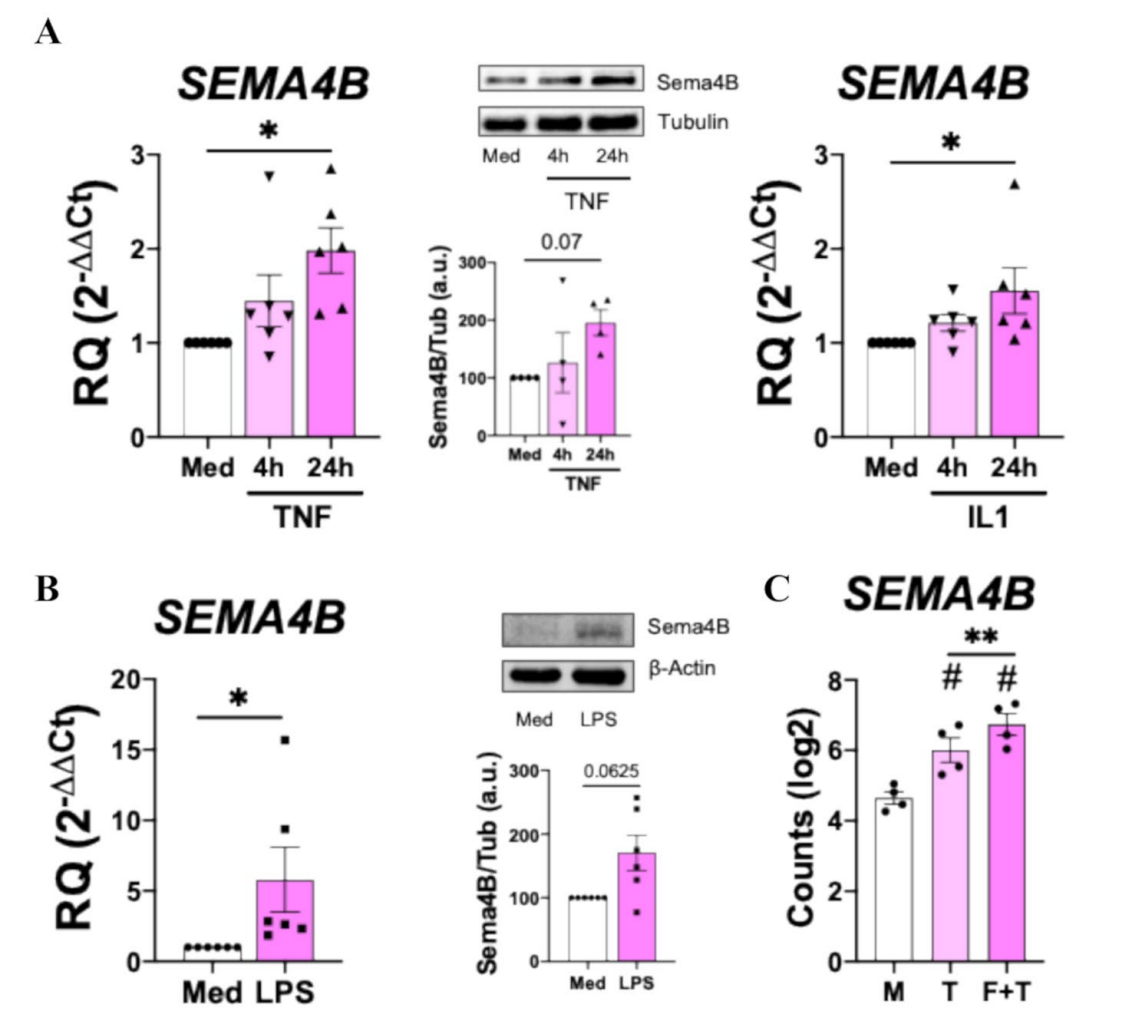
Sema4B expression induced by inflammatory mediators. **(A-B)** mRNA expression, densitometric analysis and representative immunoblot of Sema4B in RA FLS (*n* = 4–6) stimulated with TNF [10 ng/mL] or IL-1B (1 ng/mL) for 4 and 24 h (A) and RA MØ (*n* = 6) stimulated with LPS [10 ng/mL] for 24 h **(B)**. Data are shown as RQ (relative quantity, 2^− ΔΔCt^) respect to unstimulated cells or arbitrary units (A.U.). **(C)***SEMA4B* expression in RA macrophages (*n* = 4) stimulated with TNF, in the presence or absence of RA FLS, for 24 h. Data are presented as log2 of counts (Transcripts Per Kilobase Million). *p < 0.05 and **p < 0.01

We next determined whether the stimulation of RA FLS and RA MØ with key inflammatory mediators in RA pathogenesis [25] modulated the expression of Sema4B and its receptors. In RA FLS, TNF-α and IL1-β stimulation upregulated the mRNA and protein expression of Sema4B (Fig. 4A-C). In contrast, TNF-α or IL1β stimulation had no effect on the expression of *SEMA4B* receptors (Supplementary Figure S3). In RA MØ, TLR4 activation triggered by LPS ligation, induced the mRNA and protein expression of Sema4B (Fig. 4D-E). Moreover, data from a public RNAseq database showed that TNF stimulation induces the expression of *SEMA4B* by RA macrophages and this expression is further enhanced in macrophages cocultured with RA FLS and stimulated with TNF (Fig. 4F). Regarding Sema4B receptors, LPS also upregulated the expression of *DLG4*, while *PLXNB2* and *DCBLD2* remained unaffected (Supplementary Figure S4).

Altogether these data demonstrate that Sema4B signalling is elevated in RA and is associated with the inflammatory environment of the RA synovium.

### Sema4B contributes to migration and the expression of Proinflammatory cytokines by RA FLS and macrophages

**Fig. 5:**
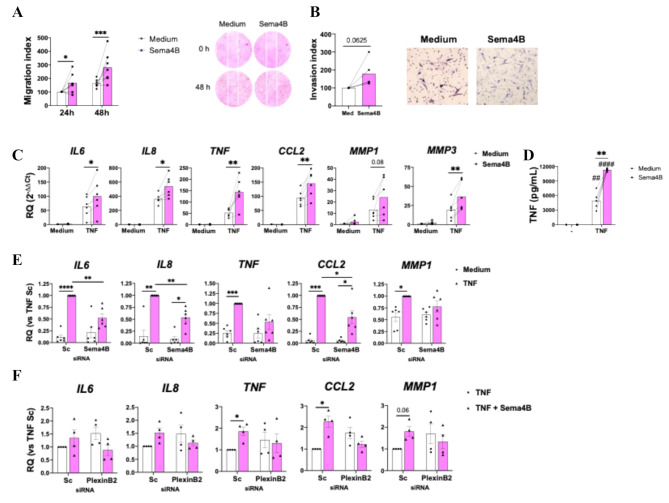
Sema4B contributes to the RA FLS invasiveness and the expression of proinflammatory cytokines. **(A-B)** Migration (*n* = 7, A) and invasion (*n* = 5, B) quantification and representative migration image of RA FLS after rhSema4B [200 ng/mL] stimulation for 24 h (A) and 48 h (A, B). **(C-D)** mRNA (C) and protein secretion (D) of proinflammatory cytokines in RA FLS stimulated with rhSema4B [200 ng/mL] in the presence or absence of TNF [10 ng/mL] for 24 h (*n* = 6). **(E)** mRNA expression of pro-inflammatory cytokines in RA FLS transfected with Sc or Sema4B siRNA and stimulated with TNF [10 ng/mL] for 24 h (*n* = 6). Data are shown as RQ (relative quantity) or percentage respect to TNF stimulated cells. **(E)** mRNA expression of pro-inflammatory cytokines in RA FLS transfected with Sc or PlexinB2 siRNA and stimulated with rhSema4B [200 ng/mL] in the presence or absence of TNF [10 ng/mL] for 24 h. Data are shown as RQ (relative quantity) or percentage respect to TNF stimulated cells. Means and SEM are shown. *p < 0.05, ** < 0.01 and ***p < 0.001; ##*p* < 0.01 and ###*p* < 0.001 compared to medium

Next, the functional roles of Sema4B were evaluated. Sema4B has been associated with cell proliferation and migration/invasion [8, 10], then we first assessed both processes in RA FLS. Sema4B did not affect RA FLS proliferation (Supplementary Figure S5), but it did induce cell migration after 24 and 48 h of stimulation (Fig. 5A) and showed a trend towards an invasiveness FLS phenotype (Fig. 5B).

The effect of Sema4B on the expression of mediators involved in RA pathogenesis was also determined. In RA FLS, Sema4B alone did not modulate the mRNA expression of any of the inflammatory mediators analysed, but it enhanced the TNF-α induced expression of *IL6*,* TNF*,* IL8*, *CCL2*, *MMP1* and *MMP3(*Fig. 5C*).* We validated this finding at the protein level, as Sema4B significantly enhanced the TNF-α induced secretion of TNF-α (Fig. 5D). Importantly, the silencing of Sema4B reduced the TNF-induced expression of inflammatory mediators, mainly IL6, IL8 and CCL2 (Fig. 5E).

Since PlexinB2 showed the highest expression among the Sema4B receptors and the levels were higher in RA FLS compared RA FLS, the functional effect of PlexinB2 silencing was also analysed. Similarly to non-transfected RA FLS, Sema4B enhanced the TNF-mediated up-regulation of inflammatory mediators in Sc siRNA transfected cells. However, the silencing of PlexinB2 abrogated this effect (Fig. 5F).

These results demonstrate a pathogenic role of Sema4B in RA FLS that is mediated, at least in part, by PlexinB2.

### Sema4B contributes to the expression of Proinflammatory cytokines in a 3D synovium model

**Fig. 6:**
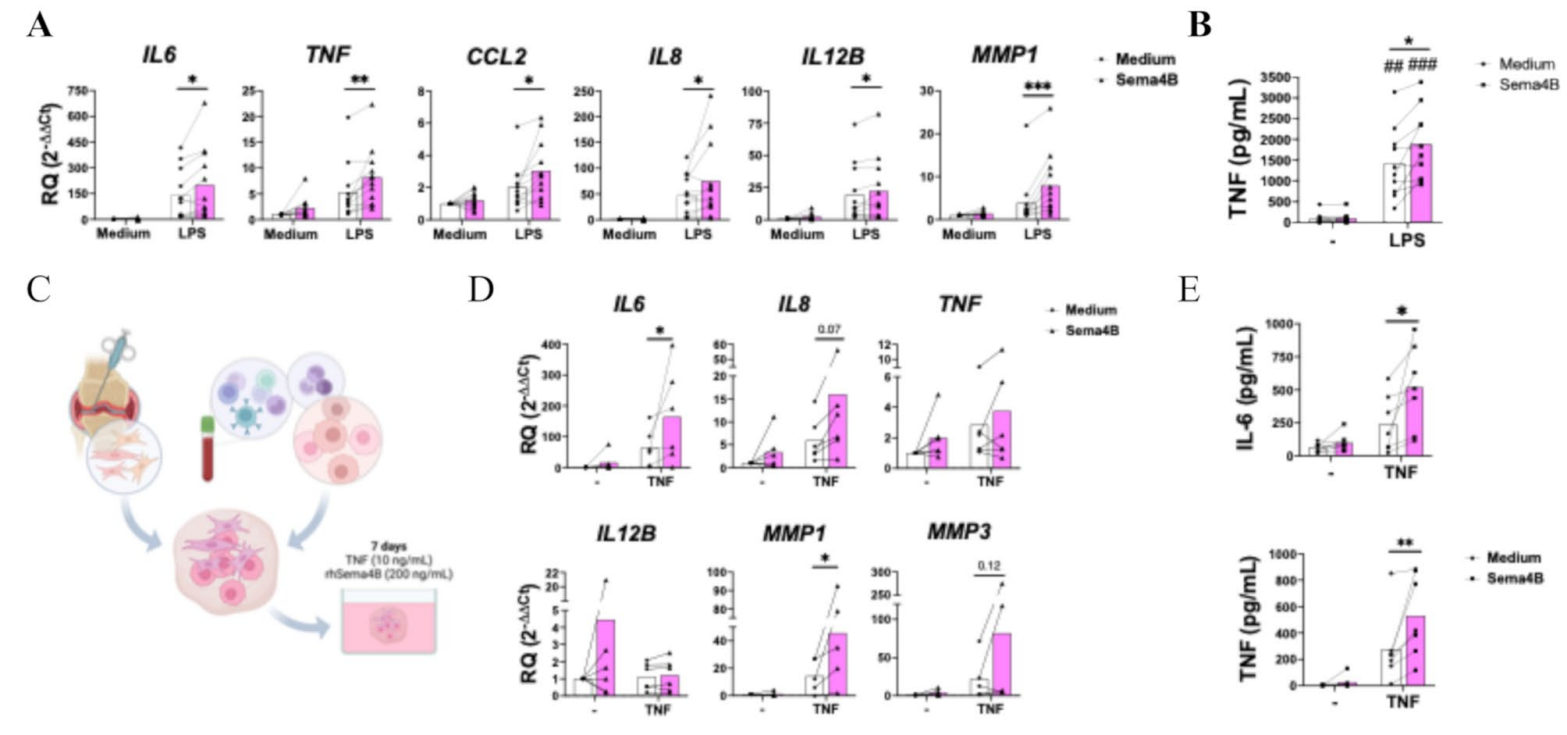
Sema4B contributes to migration and the expression of proinflammatory cytokines by RA macrophages and 3D micromasses. **(A-B)** mRNA **(A)** and protein **(B)** expression of pro-inflammatory cytokines in RA MØ stimulated with rhSema4B [200 ng/mL] in the presence or absence of LPS [10 ng/mL] for 24 h (*n* = 10). **(C)** Schematic overview of 3D micromasses synthesis. **(D-E)** mRNA **(D)** and protein **(E)** expression of pro-inflammatory cytokines in the 3D micromasses after stimulation with rhSema4B [200 ng/mL] in the presence or absence of TNF [10 ng/mL] for 7d (*n* = 6–7). Means and SEM are shown. Data are shown as RQ (relative quantity) respect to unstimulated cells. *p < 0.05, **p < 0.01 and ***p < 0.001; ##*p* < 0.01 and ###*p* < 0.001 compared to medium

Due to the relevance of MØ in promoting inflammation in RA pathogenesis, we also determined the effect of Sema4B on RA MØ. Similarly to FLS, Sema4B alone did not have any inflammatory effect, but significantly enhanced the LPS-mediated expression of *IL6*, *IL8*, *IL12B*, *MMP1*, *CCL2* and *TNF(*Fig. 6A*)*. Moreover, Sema4B significantly increased the LPS-induced protein secretion of TNF-α (Fig. 6B).

To validate the inflammatory effect of Sema4B in a more complex system, we finally tested it on 3D synovium micromasses constituted by RA FLS and RA CD14^+^ monocytes (Fig. 6C). The presence of Sema4B during the formation of the 3D synovium induced the expression of *IL8*, *IL12B* and *TNF*, although differences were not significant. In line with the previous findings, Sema4B significantly enhanced the TNF-induced expression of *IL6*,* IL8*,* MMP1* and *MMP3*, as well as the secretion of IL-6 and TNF (Fig. 6E).

Overall, these findings demonstrate the contribution of Sema4B to the inflammatory and destructive environment observed within the RA synovium.

## Discussion

In this work we have identified Sema4B as another member of the semaphorin family involved in the pathogenesis of RA. Concretely, Sema4B displays an inflammatory and destructive role that is linked to the boosting of pathogenic phenotypes of FLS and MØ, the main cells constituting the synovial membrane within a context of RA [26]. This aligns with the results of earlier studies about the class 4 semaphorins [5, 6, 27], supporting the premise that these proteins are active collaborators in the pathogenic pathways of RA.

Firstly, it was found that Sema4B is elevated in the synovial tissue of patients with RA compared to HC. Interestingly, the expression of Sema4B was higher in the inflamed synovium than in the non-inflamed tissue and positively correlated with clinical disease parameters and the expression of inflammatory mediators. These findings, in combination with the TNF-α and IL1-β induced Sema4B expression by FLS, suggest that these mediators, which are highly expressed in the synovium of patients with RA [28], are responsible of the elevated Sema4B expression. On their part, TLR4 activation induced MØ Sema4B expression, suggesting that endogenous synovial TLR ligands, such as heat- shock proteins and fibronectin [28], may be also involved in the elevated expression of Sema4B. PlexinB2, the most studied receptor for Sema4B, is also elevated in the inflamed synovium of patients with RA and in RA FLS.

Secondly, we demonstrated the pathogenic roles of Sema4B in RA. In line with other cell types [8], Sema4B induced RA FLS migration and invasion, which may contribute to the aggressive and destructive phenotype of this cell type [26]. Regarding the inflammatory role in RA FLS and MØ, Sema4B alone did not have any effect, but enhanced the expression of pro-inflammatory genes induced by TNF and LPS, respectively. A recent work of Casden N and colleagues showed similar results, since inflammatory effect of Sema4B in microglia/macrophages is only observed in the presence of an inflammatory environment [11]. This is not an unusual finding in macrophages or FLS; in fact, TNF can sensitize macrophages to type I interferon (IFN) or Tie2 signaling [29, 30] and enhances responses (priming) to IFN challenge in RA FLS, promoting their subsequent inflammatory response to IFNs [31]. Finally, the inflammatory role of Sema4B in a 3D synovium model support this aim. As expected, based on the *in vitro* experiments, Sema4B enhanced the TNF-induced expression of inflammatory mediators. Interestingly, Sema4B alone was able to induce, although in a lower extent, the expression of inflammatory mediators. Then, inflammatory mediators secreted by M1 differentiated MØ could prime RA FLS to Sema4B stimulation.

The inflammatory effect of Sema4B in RA FLS was mediated by PlexinB2. This receptor is also involved in the Sema4B-mediated inflammation in microglia/macrophages [11], the Sema4D-mediated cartilage destruction [7], the Sema4A-induced secretion of IL-17 by CD4^+^ T cells of systemic sclerosis patients [32] and the Sema4D-mediated inflammation in a mouse model of multiple sclerosis [33]. As these PlexinB2-mediated effects are key processes in the pathogenesis of RA, and the expression of these semaphorins and PlexinB2 is elevated in the synovium of patients with RA, it is tempting to speculate that PlexinB2 might be a potential therapeutic target in the treatment of this disease.

A limitation of this study is that we used in vitro differentiated MØ instead RA synovial macrophages, limiting the strength of our findings. Another issue is the qPCR analysis in the micromasses, since we cannot compare the single effect of Sema4B in MØ and FLS. Therefore, effect on sorted populations need to be performed for elucidating this effect.

Overall, this work postulates Sema4B as a new mediator involved in the inflammatory processes observed in patients with RA, pointing out the importance of class 4 semaphorins and PlexinB2 signalling in the pathogenesis of this disease.

## Electronic supplementary material

Below is the link to the electronic supplementary material.

Supplementary Material 1

## Data Availability

No datasets were generated or analysed during the current study.
