# Supplementary material for: Semaphorin4B is elevated in rheumatoid arthritis and enhances the inflammatory phenotype of macrophages and fibroblast-like synoviocytes
Source: Arthritis Res Ther. 2025 Jul 1;27:132. doi: 10.1186/s13075-025-03592-x (PMC12219977; doi:10.1186/s13075-025-03592-x)
Supplement: Supplementary file 1 — Supplementary Material 1 [file 13075_2025_3592_MOESM1_ESM.pdf]

Supplementary methods

Supplementary tables

Supplementary Table S1. Characteristics of RA patients at the time of blood sampling. Data is presented as the median (interquartile range) or number (percentage). RF: rheumatoid factor; ACPA: Anti-citrullinated protein antibodies; ESR: erythrocyte sedimentation rate; CRP: C-reactive protein; DAS28: Disease Activity Score 28; NSAIDs: non-steroidal anti-inflammatory drugs; DMARDs: disease-modifying antirheumatic drugs.

	**RA (n = 26)**
**Age (years)**	63 (49 – 71)
**Female: n (%)**	15 (57.7 %)
**RF^{+}**	18 (69.2 %)
**$ACPA^{+}$**	17 (65.4 %)
**ESR (mm/Hr)**	16.0 (6 – 43.2)
**CRP (mg/L)**	6.8 (5.15 – 12.9)
**DAS28**	4.29 (2.8 – 5.1)
**NSAIDs**	3 (11.5 %)
**csDMARDs**	18 (69.2 %)
**bDMARDs**	4 (15.4 %)

Supplementary Table S2. List of primers. Length data in base pair

Gene	Primer forward	Primer reverse
B2M	GATGAGTATGCCTGCCGTGT	TGCGGCATCTTCAAACCTCC
CCL2	TCTGTGCCTGCTGCTCATAG	GGGCATTGATTGCATCTGGC
DCBLD2	ATGTGGACACACTGTACTAGGC	CTGTTGGGATAGGTCTGTGGG
DLG4	TCGGTGACGACCCATCCAT	GCACGTCCACTTCATTTACAAAC
GAPDH	GCCAGCCGAGCCACATC	TGACCAGGCGCCCAATAC
IL12B	ACGTTTCACCTGCTGGTGGCT	CTCCGCACGTCACCCCTTGG
IL6	GACAGCCACTCACCTCTTCA	CCTCTTTGCTGCTTTCACAC
IL8	GCTCTGTGTGAAGGTGCAGT	CCAGACAGAGCTCTCTTCCA
MMP1	CGGGGCTTTGATGTACCCTA	CGATGGGCTGGACAGGATTT
MMP3	GAGGACACCAGCATGAACCT	CACCTCCAGAGTGTCGGAGT
PLXB1	CTGCAGAAGTTCGTGGATGA	GAGGCAAGCTGTTGGTCTTC
RPL13	CCTGGAGGAGAAGAGGAAAGAGA	TTGAGGACCTCTGTGTATTTGTCAA
SEMA4B	CTTCAGCCCCATGTGTACCT	CAGTGTAGAGCTCGCCATCA
TNF	TCTTCTCGAACCCCGAGTGA	CCTCTGATGGCACCACCAG

Supplementary figures

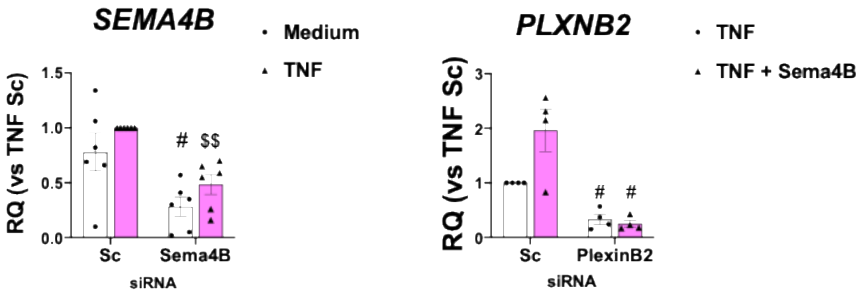

Supplementary Figure S1. Efficiency of cell transfection in RA FLS. mRNA expression of Sema4B and PlexinB2 in RA FLS transfected with scrambled control (Sc), Sema4B siRNA or PlexinB2 siRNA. Data is shown as relative quantity (RQ) respect to Sc siRNA-transfected cells stimulated with TNF [10 ng/mL] for 24 h (n=4-6). Means and SEM are shown. #p < 0.05, compared to Sc medium or Sc TNF, $$ p < 0.01, compared to Sc medium or Sc TNF.

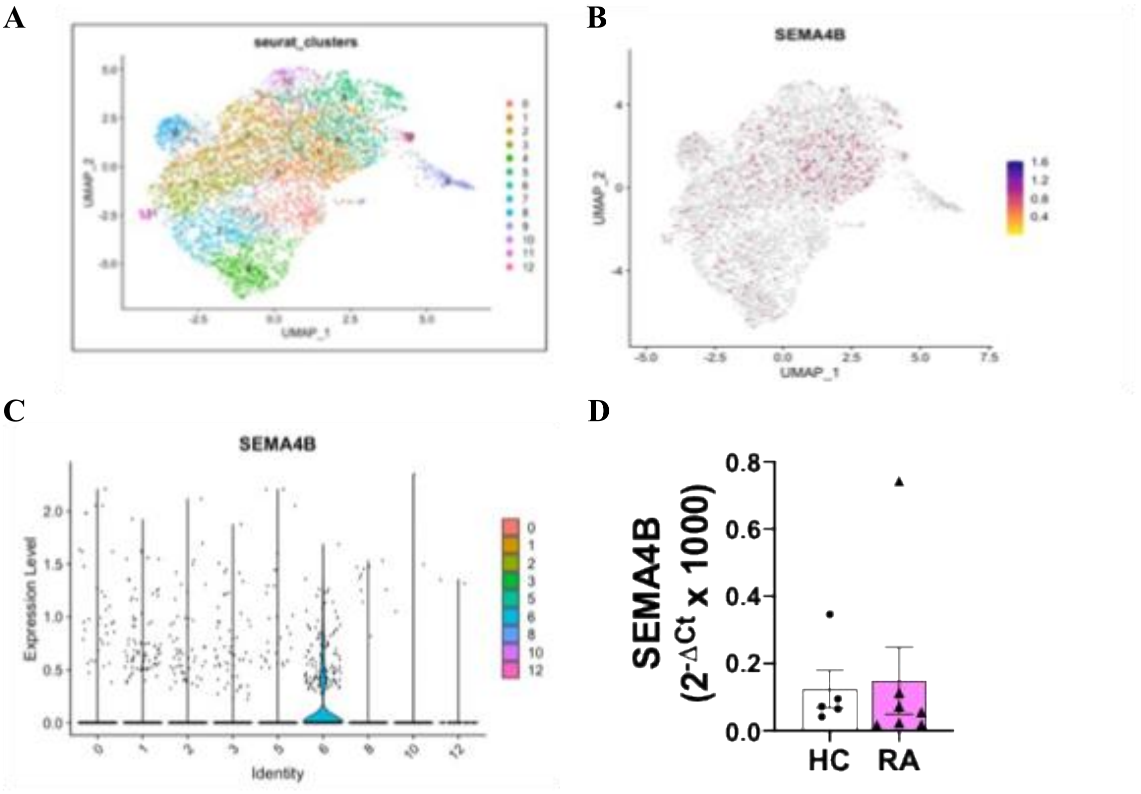

Figure 2. Sema4B is expressed by macrophages populations. (A) High dimensionality single cell RNA sequencing analysis identifies specific macrophage clusters in patient with RA synovial tissue biopsies. **(B)** Feature plots for the expression and distribution of *SEMA4B* in all cells**. (C)** Expression positive cells per macrophage cluster for *SEMA4B*. **(D)** mRNA expression of Sema4B in RA and HC macrophages (n=5-7). Means and SEM are shown.

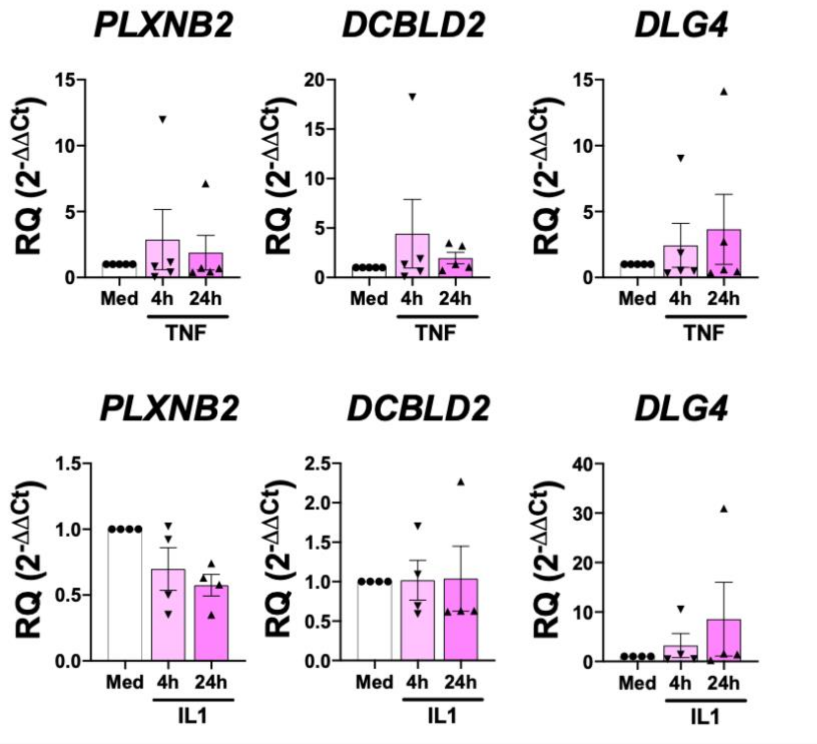

Supplementary Figure S3. Sema4B receptors expression in stimulated RA FLS. mRNA expression of Sema4B receptors in RA FLS stimulated with TNF [10 ng/mL] or IL1 [1 ng/mL] for 4 and 24 h (n=4-5). Data are shown as RQ (relative quantity) respect to unstimulated cells. Means and SEM are shown.

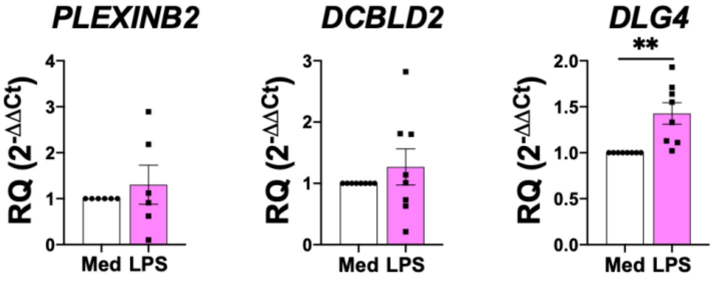

Supplementary Figure S4. Sema4B receptors expression in stimulated macrophages. mRNA expression of Sema4B receptors in RA macrophages stimulated with LPS [10 ng/mL] for 4 (n=6-8). Data are shown as RQ (relative quantity) respect to unstimulated cells. Means and SEM are shown. * $p < 0.05$.

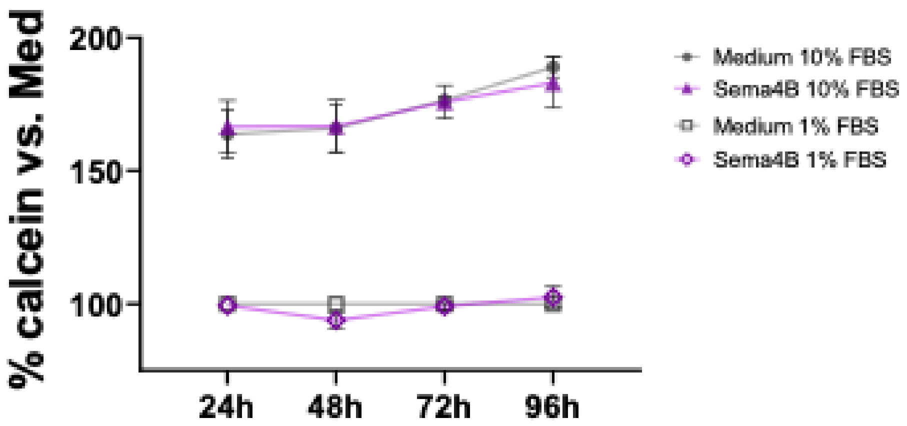

Supplementary Figure S5. Sema4B does not affect to RA FLS viability. Viability of RA FLS after rhSema4B [200 ng/mL] stimulation at the indicated time points. Means and SEM are shown.

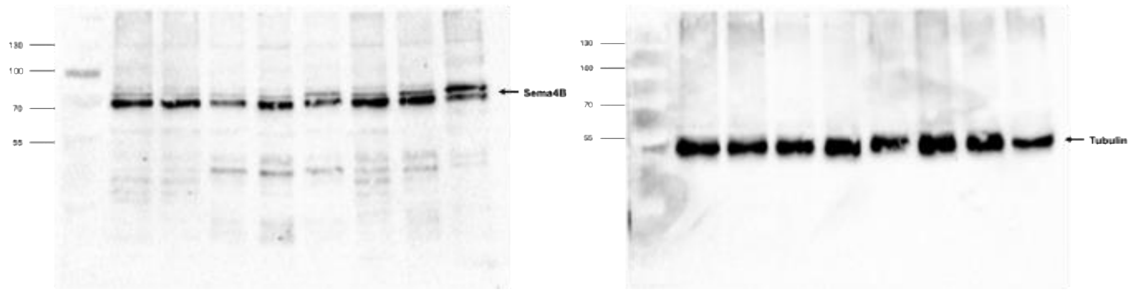

Supplementary Figure S7. Uncropped blots shown in Figure 1C.

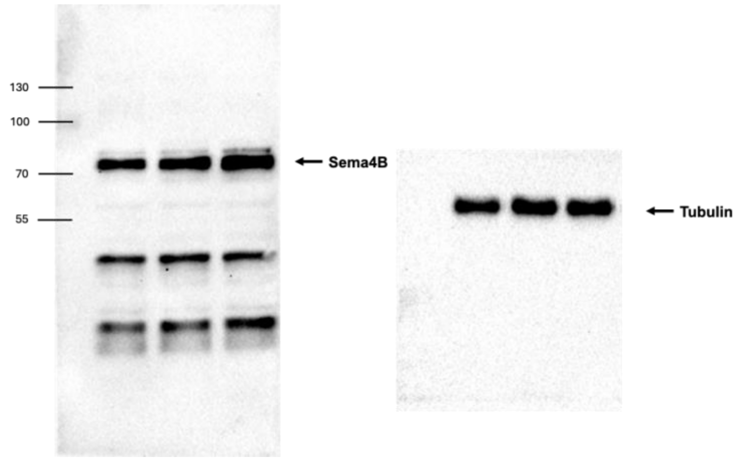

Supplementary Figure S8. Uncropped blots shown in Figure 2A.

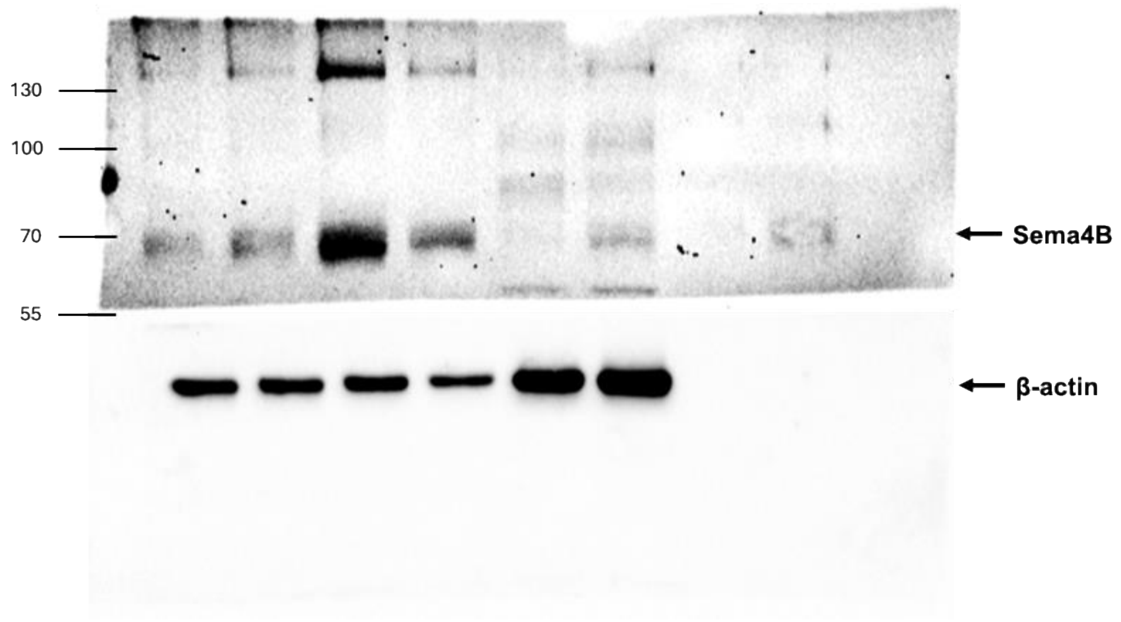

Supplementary Figure S9. Uncropped blots shown in Figure 2C.
